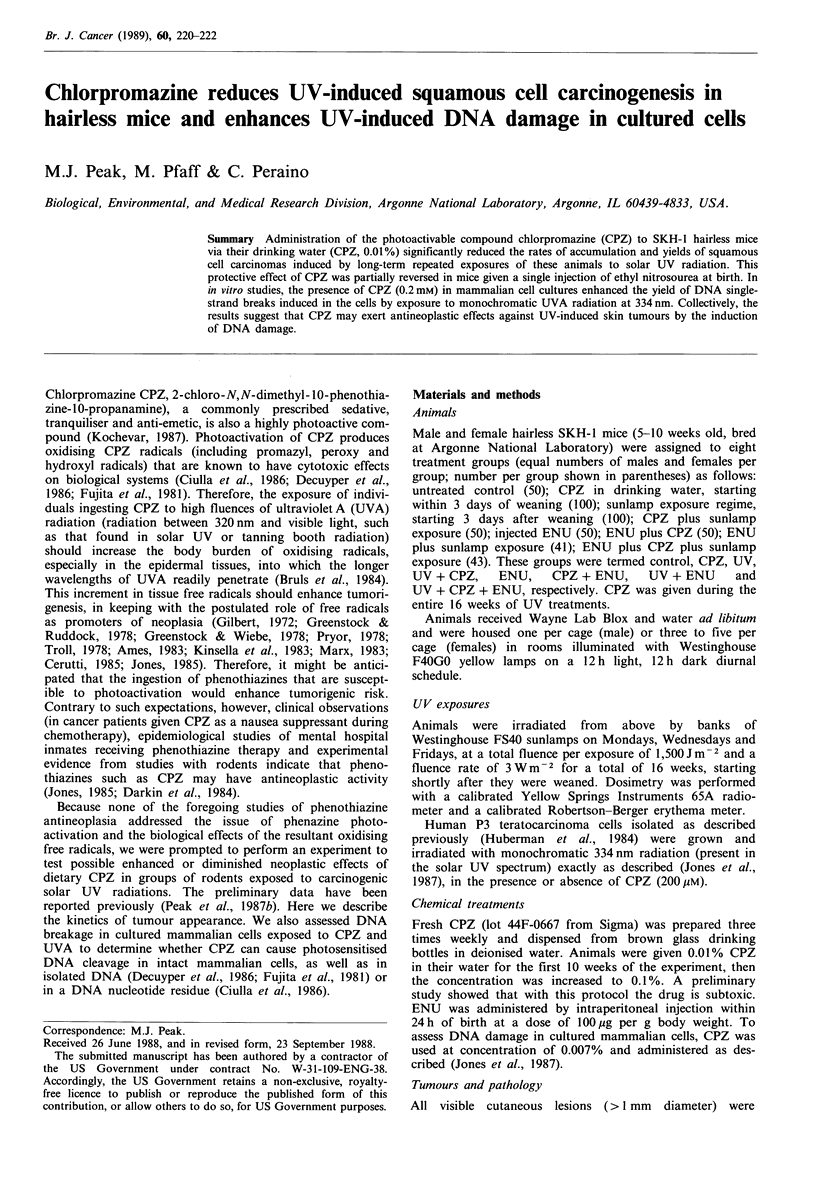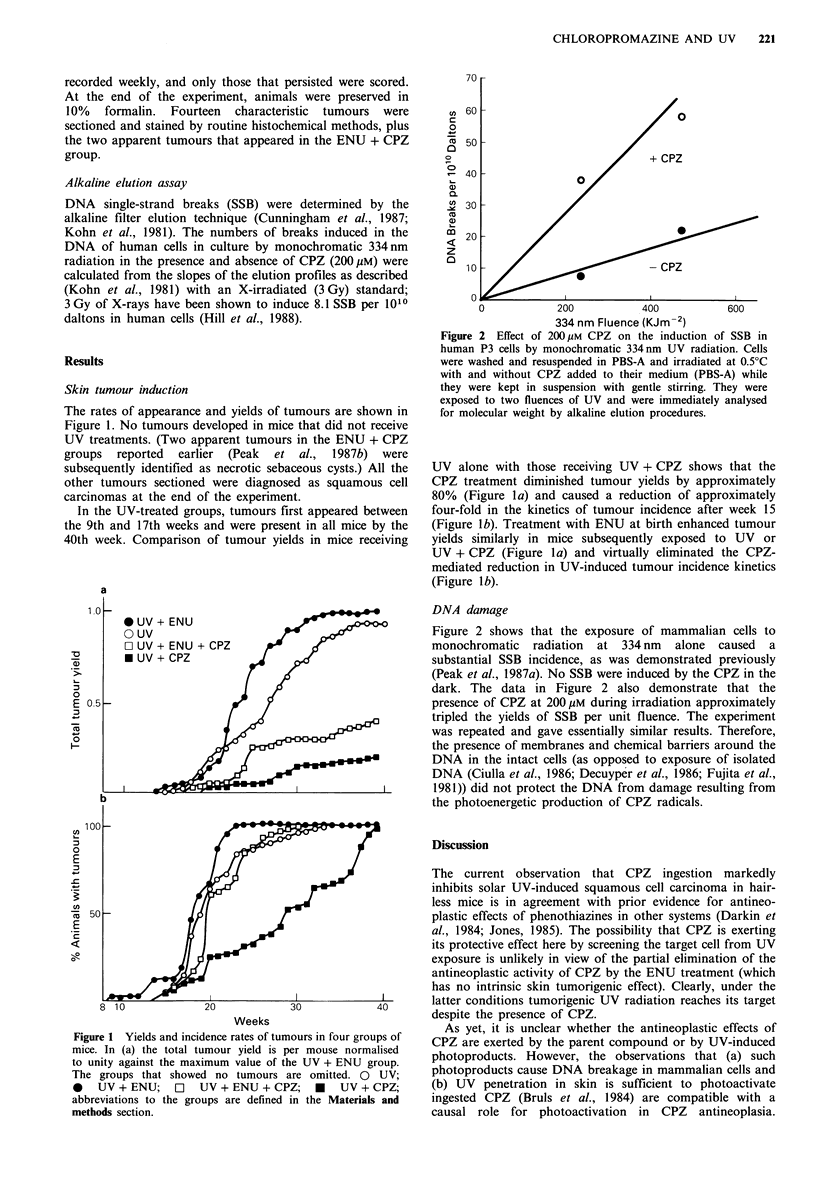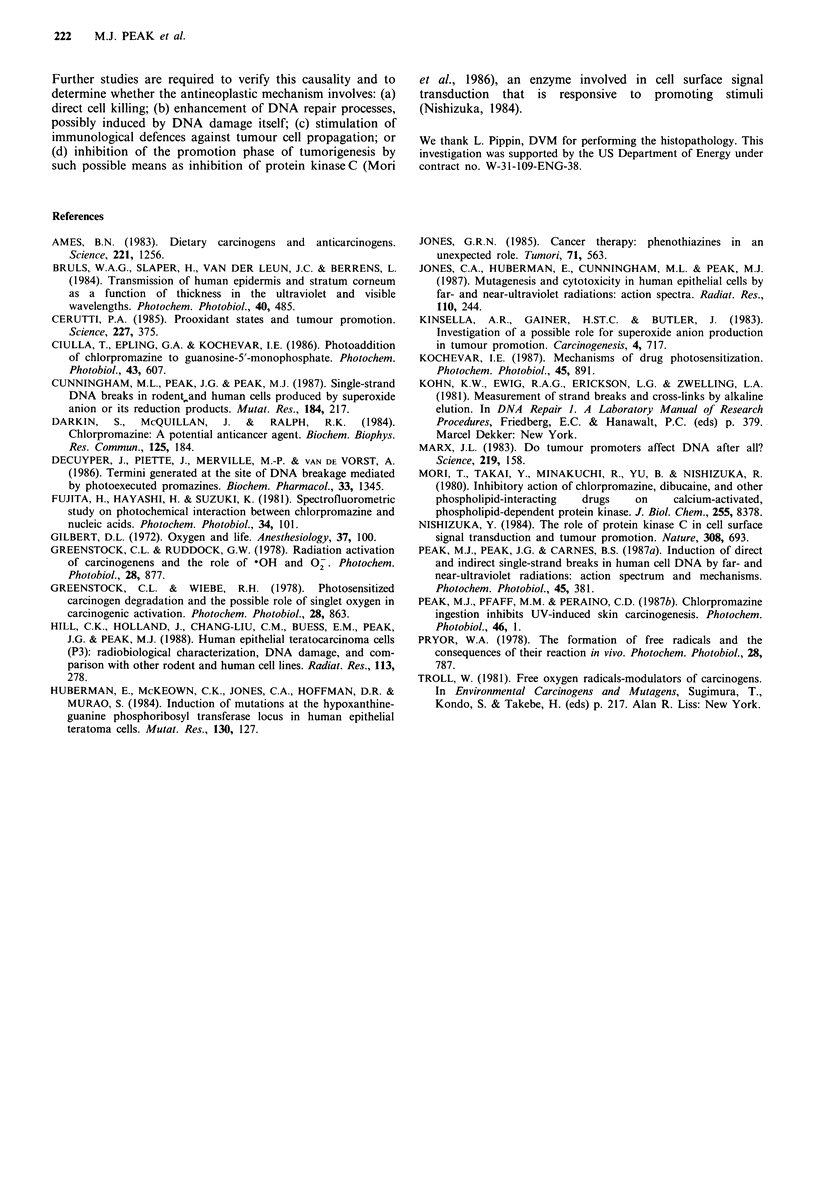# Chlorpromazine reduces UV-induced squamous cell carcinogenesis in hairless mice and enhances UV-induced DNA damage in cultured cells.

**DOI:** 10.1038/bjc.1989.255

**Published:** 1989-08

**Authors:** M. J. Peak, M. Pfaff, C. Peraino

**Affiliations:** Biological, Environmental, and Medical Research Division, Argonne National Laboratory, IL 60439-4833.

## Abstract

Administration of the photoactivable compound chlorpromazine (CPZ) to SKH-1 hairless mice via their drinking water (CPZ, 0.01%) significantly reduced the rates of accumulation and yields of squamous cell carcinomas induced by long-term repeated exposures of these animals to solar UV radiation. This protective effect of CPZ was partially reversed in mice given a single injection of ethyl nitrosourea at birth. In in vitro studies, the presence of CPZ (0.2 mM) in mammalian cell cultures enhanced the yield of DNA single-strand breaks induced in the cells by exposure to monochromatic UVA radiation at 334 nm. Collectively, the results suggest that CPZ may exert antineoplastic effects against UV-induced skin tumours by the induction of DNA damage.


Br. J. Cancer (1989), 60, 220–222

### Chlorpromazine reduces UV-induced squamous cell carcinogenesis in hairless mice and enhances UV-induced DNA damage in cultured cells

M.J. Peak, M. Pfaff & C. Peraino

*Biological, Environmental, and Medical Research Division, Argonne National Laboratory, Argonne, IL 60439-4833, USA.*

**Summary** Administration of the photoactivable compound chlorpromazine (CPZ) to SKH-1 hairless mice via their drinking water (CPZ, 0.01%) significantly reduced the rates of accumulation and yields of squamous cell carcinomas induced by long-term repeated exposures of these animals to solar UV radiation. This protective effect of CPZ was partially reversed in mice given a single injection of ethyl nitrosourea at birth. In *in vitro* studies, the presence of CPZ (0.2 mM) in mammalian cell cultures enhanced the yield of DNA single-strand breaks induced in the cells by exposure to monochromatic UVA radiation at 334 nm. Collectively, the results suggest that CPZ may exert antineoplastic effects against UV-induced skin tumours by the induction of DNA damage.

Chlorpromazine CPZ, 2-chloro-*N*,*N*-dimethyl-10-phenothiazine-10-propanamine), a commonly prescribed sedative, tranquiliser and anti-emetic, is also a highly photoactive compound (Kochevar, 1987). Photoactivation of CPZ produces oxidising CPZ radicals (including promazyl, peroxy and hydroxyl radicals) that are known to have cytotoxic effects on biological systems (Ciulla *et al.*, 1986; Decuyper *et al.*, 1986; Fujita *et al.*, 1981). Therefore, the exposure of individuals ingesting CPZ to high fluences of ultraviolet A (UVA) radiation (radiation between 320 nm and visible light, such as that found in solar UV or tanning booth radiation) should increase the body burden of oxidising radicals, especially in the epidermal tissues, into which the longer wavelengths of UVA readily penetrate (Bruls *et al.*, 1984). This increment in tissue free radicals should enhance tumorigenesis, in keeping with the postulated role of free radicals as promoters of neoplasia (Gilbert, 1972; Greenstock & Ruddock, 1978; Greenstock & Wiebe, 1978; Pryor, 1978; Troll, 1978; Ames, 1983; Kinsella *et al.*, 1983; Marx, 1983; Cerutti, 1985; Jones, 1985). Therefore, it might be anticipated that the ingestion of phenothiazines that are susceptible to photoactivation would enhance tumorigenic risk. Contrary to such expectations, however, clinical observations (in cancer patients given CPZ as a nausea suppressant during chemotherapy), epidemiological studies of mental hospital inmates receiving phenothiazine therapy and experimental evidence from studies with rodents indicate that phenothiazines such as CPZ may have antineoplastic activity (Jones, 1985; Darkin *et al.*, 1984).

Because none of the foregoing studies of phenothiazine antineoplasia addressed the issue of phenazine photoactivation and the biological effects of the resultant oxidising free radicals, we were prompted to perform an experiment to test possible enhanced or diminished neoplastic effects of dietary CPZ in groups of rodents exposed to carcinogenic solar UV radiations. The preliminary data have been reported previously (Peak *et al.*, 1987*b*). Here we describe the kinetics of tumour appearance. We also assessed DNA breakage in cultured mammalian cells exposed to CPZ and UVA to determine whether CPZ can cause photosensitised DNA cleavage in intact mammalian cells, as well as in isolated DNA (Decuyper *et al.*, 1986; Fujita *et al.*, 1981) or in a DNA nucleotide residue (Ciulla *et al.*, 1986).

Correspondence: M.J. Peak.
Received 26 June 1988, and in revised form, 23 September 1988.

The submitted manuscript has been authored by a contractor of the US Government under contract No. W-31-109-ENG-38. Accordingly, the US Government retains a non-exclusive, royalty-free licence to publish or reproduce the published form of this contribution, or allow others to do so, for US Government purposes.

### Materials and methods

### *Animals*

Male and female hairless SKH-1 mice (5–10 weeks old, bred at Argonne National Laboratory) were assigned to eight treatment groups (equal numbers of males and females per group; number per group shown in parentheses) as follows: untreated control (50); CPZ in drinking water, starting within 3 days of weaning (100); sunlamp exposure regime, starting 3 days after weaning (100); CPZ plus sunlamp exposure (50); injected ENU (50); ENU plus CPZ (50); ENU plus sunlamp exposure (41); ENU plus CPZ plus sunlamp exposure (43). These groups were termed control, CPZ, UV, UV + CPZ, ENU, CPZ + ENU, UV + ENU and UV + CPZ + ENU, respectively. CPZ was given during the entire 16 weeks of UV treatments.

Animals received Wayne Lab Blox and water *ad libitum* and were housed one per cage (male) or three to five per cage (females) in rooms illuminated with Westinghouse F40G0 yellow lamps on a 12 h light, 12 h dark diurnal schedule.

### *UV exposures*

Animals were irradiated from above by banks of Westinghouse FS40 sunlamps on Mondays, Wednesdays and Fridays, at a total fluence per exposure of 1,500 $J m^{-2}$ and a fluence rate of 3 $W m^{-2}$ for a total of 16 weeks, starting shortly after they were weaned. Dosimetry was performed with a calibrated Yellow Springs Instruments 65A radiometer and a calibrated Robertson–Berger erythema meter.

Human P3 teratocarcinoma cells isolated as described previously (Huberman *et al.*, 1984) were grown and irradiated with monochromatic 334 nm radiation (present in the solar UV spectrum) exactly as described (Jones *et al.*, 1987), in the presence or absence of CPZ (200 $\mu$M).

### *Chemical treatments*

Fresh CPZ (lot 44F-0667 from Sigma) was prepared three times weekly and dispensed from brown glass drinking bottles in deionised water. Animals were given 0.01% CPZ in their water for the first 10 weeks of the experiment, then the concentration was increased to 0.1%. A preliminary study showed that with this protocol the drug is subtoxic. ENU was administered by intraperitoneal injection within 24 h of birth at a dose of 100 $\mu$g per g body weight. To assess DNA damage in cultured mammalian cells, CPZ was used at concentration of 0.007% and administered as described (Jones *et al.*, 1987).

### *Tumours and pathology*

All visible cutaneous lesions (>1 mm diameter) were recorded weekly, and only those that persisted were scored. At the end of the experiment, animals were preserved in 10% formalin. Fourteen characteristic tumours were sectioned and stained by routine histochemical methods, plus the two apparent tumours that appeared in the ENU + CPZ group.

*Alkaline elution assay*

DNA single-strand breaks (SSB) were determined by the alkaline filter elution technique (Cunningham *et al.*, 1987; Kohn *et al.*, 1981). The numbers of breaks induced in the DNA of human cells in culture by monochromatic 334 nm radiation in the presence and absence of CPZ (200 $\mu$M) were calculated from the slopes of the elution profiles as described (Kohn *et al.*, 1981) with an X-irradiated (3 Gy) standard; 3 Gy of X-rays have been shown to induce 8.1 SSB per $10^{10}$ daltons in human cells (Hill *et al.*, 1988).

### Results

*Skin tumour induction*

The rates of appearance and yields of tumours are shown in Figure 1. No tumours developed in mice that did not receive UV treatments. (Two apparent tumours in the ENU + CPZ groups reported earlier (Peak *et al.*, 1987*b*) were subsequently identified as necrotic sebaceous cysts.) All the other tumours sectioned were diagnosed as squamous cell carcinomas at the end of the experiment.

In the UV-treated groups, tumours first appeared between the 9th and 17th weeks and were present in all mice by the 40th week. Comparison of tumour yields in mice receiving UV alone with those receiving UV + CPZ shows that the CPZ treatment diminished tumour yields by approximately 80% (Figure 1*a*) and caused a reduction of approximately four-fold in the kinetics of tumour incidence after week 15 (Figure 1*b*). Treatment with ENU at birth enhanced tumour yields similarly in mice subsequently exposed to UV or UV + CPZ (Figure 1*a*) and virtually eliminated the CPZ-mediated reduction in UV-induced tumour incidence kinetics (Figure 1*b*).

**Figure 1** Yields and incidence rates of tumours in four groups of mice. In (a) the total tumour yield is per mouse normalised to unity against the maximum value of the UV + ENU group. The groups that showed no tumours are omitted. ○ UV; ● UV + ENU; □ UV + ENU + CPZ; ■ UV + CPZ; abbreviations to the groups are defined in the **Materials and methods** section.

**Figure 2** Effect of 200 $\mu$M CPZ on the induction of SSB in human P3 cells by monochromatic 334 nm UV radiation. Cells were washed and resuspended in PBS-A and irradiated at 0.5°C with and without CPZ added to their medium (PBS-A) while they were kept in suspension with gentle stirring. They were exposed to two fluences of UV and were immediately analysed for molecular weight by alkaline elution procedures.

*DNA damage*

Figure 2 shows that the exposure of mammalian cells to monochromatic radiation at 334 nm alone caused a substantial SSB incidence, as was demonstrated previously (Peak *et al.*, 1987*a*). No SSB were induced by the CPZ in the dark. The data in Figure 2 also demonstrate that the presence of CPZ at 200 $\mu$M during irradiation approximately tripled the yields of SSB per unit fluence. The experiment was repeated and gave essentially similar results. Therefore, the presence of membranes and chemical barriers around the DNA in the intact cells (as opposed to exposure of isolated DNA (Ciulla *et al.*, 1986; Decuyper *et al.*, 1986; Fujita *et al.*, 1981)) did not protect the DNA from damage resulting from the photoenergetic production of CPZ radicals.

### Discussion

The current observation that CPZ ingestion markedly inhibits solar UV-induced squamous cell carcinoma in hairless mice is in agreement with prior evidence for antineoplastic effects of phenothiazines in other systems (Darkin *et al.*, 1984; Jones, 1985). The possibility that CPZ is exerting its protective effect here by screening the target cell from UV exposure is unlikely in view of the partial elimination of the antineoplastic activity of CPZ by the ENU treatment (which has no intrinsic skin tumorigenic effect). Clearly, under the latter conditions tumorigenic UV radiation reaches its target despite the presence of CPZ.

As yet, it is unclear whether the antineoplastic effects of CPZ are exerted by the parent compound or by UV-induced photoproducts. However, the observations that (a) such photoproducts cause DNA breakage in mammalian cells and (b) UV penetration in skin is sufficient to photoactivate ingested CPZ (Bruls *et al.*, 1984) are compatible with a causal role for photoactivation in CPZ antineoplasia.

Further studies are required to verify this causality and to determine whether the antineoplastic mechanism involves: (a) direct cell killing; (b) enhancement of DNA repair processes, possibly induced by DNA damage itself; (c) stimulation of immunological defences against tumour cell propagation; or (d) inhibition of the promotion phase of tumorigenesis by such possible means as inhibition of protein kinase C (Mori *et al.*, 1986), an enzyme involved in cell surface signal transduction that is responsive to promoting stimuli (Nishizuka, 1984).

We thank L. Pippin, DVM for performing the histopathology. This investigation was supported by the US Department of Energy under contract no. W-31-109-ENG-38.

### References

AMES, B.N. (1983). Dietary carcinogens and anticarcinogens. *Science*, **221**, 1256.

BRULS, W.A.G., SLAPER, H., VAN DER LEUN, J.C. & BERRENS, L. (1984). Transmission of human epidermis and stratum corneum as a function of thickness in the ultraviolet and visible wavelengths. *Photochem. Photobiol.*, **40**, 485.

CERUTTI, P.A. (1985). Prooxidant states and tumour promotion. *Science*, **227**, 375.

CIULLA, T., EPLING, G.A. & KOCHEVAR, I.E. (1986). Photoaddition of chlorpromazine to guanosine-5′-monophosphate. *Photochem. Photobiol.*, **43**, 607.

CUNNINGHAM, M.L., PEAK, J.G. & PEAK, M.J. (1987). Single-strand DNA breaks in rodent and human cells produced by superoxide anion or its reduction products. *Mutat. Res.*, **184**, 217.

DARKIN, S., MCQUILLAN, J. & RALPH, R.K. (1984). Chlorpromazine: A potential anticancer agent. *Biochem. Biophys. Res. Commun.*, **125**, 184.

DECUYPER, J., PIETTE, J., MERVILLE, M.-P. & VAN DE VORST, A. (1986). Termini generated at the site of DNA breakage mediated by photoexecuted promazines. *Biochem. Pharmacol.*, **33**, 1345.

FUJITA, H., HAYASHI, H. & SUZUKI, K. (1981). Spectrofluorometric study on photochemical interaction between chlorpromazine and nucleic acids. *Photochem. Photobiol.*, **34**, 101.

GILBERT, D.L. (1972). Oxygen and life. *Anesthesiology*, **37**, 100.

GREENSTOCK, C.L. & RUDDOCK, G.W. (1978). Radiation activation of carcinogenens and the role of •OH and $O_2^-$. *Photochem. Photobiol.*, **28**, 877.

GREENSTOCK, C.L. & WIEBE, R.H. (1978). Photosensitized carcinogen degradation and the possible role of singlet oxygen in carcinogenic activation. *Photochem. Photobiol.*, **28**, 863.

HILL, C.K., HOLLAND, J., CHANG-LIU, C.M., BUESS, E.M., PEAK, J.G. & PEAK, M.J. (1988). Human epithelial teratocarcinoma cells (P3): radiobiological characterization, DNA damage, and comparison with other rodent and human cell lines. *Radiat. Res.*, **113**, 278.

HUBERMAN, E., MCKEOWN, C.K., JONES, C.A., HOFFMAN, D.R. & MURAO, S. (1984). Induction of mutations at the hypoxanthine-guanine phosphoribosyl transferase locus in human epithelial teratoma cells. *Mutat. Res.*, **130**, 127.

JONES, G.R.N. (1985). Cancer therapy: phenothiazines in an unexpected role. *Tumori*, **71**, 563.

JONES, C.A., HUBERMAN, E., CUNNINGHAM, M.L. & PEAK, M.J. (1987). Mutagenesis and cytotoxicity in human epithelial cells by far- and near-ultraviolet radiations: action spectra. *Radiat. Res.*, **110**, 244.

KINSELLA, A.R., GAINER, H.ST.C. & BUTLER, J. (1983). Investigation of a possible role for superoxide anion production in tumour promotion. *Carcinogenesis*, **4**, 717.

KOCHEVAR, I.E. (1987). Mechanisms of drug photosensitization. *Photochem. Photobiol.*, **45**, 891.

KOHN, K.W., EWIG, R.A.G., ERICKSON, L.G. & ZWELLING, L.A. (1981). Measurement of strand breaks and cross-links by alkaline elution. In *DNA Repair 1. A Laboratory Manual of Research Procedures*, Friedberg, E.C. & Hanawalt, P.C. (eds) p. 379. Marcel Dekker: New York.

MARX, J.L. (1983). Do tumour promoters affect DNA after all? *Science*, **219**, 158.

MORI, T., TAKAI, Y., MINAKUCHI, R., YU, B. & NISHIZUKA, R. (1980). Inhibitory action of chlorpromazine, dibucaine, and other phospholipid-interacting drugs on calcium-activated, phospholipid-dependent protein kinase. *J. Biol. Chem.*, **255**, 8378.

NISHIZUKA, Y. (1984). The role of protein kinase C in cell surface signal transduction and tumour promotion. *Nature*, **308**, 693.

PEAK, M.J., PEAK, J.G. & CARNES, B.S. (1987*a*). Induction of direct and indirect single-strand breaks in human cell DNA by far- and near-ultraviolet radiations: action spectrum and mechanisms. *Photochem. Photobiol.*, **45**, 381.

PEAK, M.J., PFAFF, M.M. & PERAINO, C.D. (1987*b*). Chlorpromazine ingestion inhibits UV-induced skin carcinogenesis. *Photochem. Photobiol.*, **46**, 1.

PRYOR, W.A. (1978). The formation of free radicals and the consequences of their reaction *in vivo*. *Photochem. Photobiol.*, **28**, 787.

TROLL, W. (1981). Free oxygen radicals-modulators of carcinogens. In *Environmental Carcinogens and Mutagens*, Sugimura, T., Kondo, S. & Takebe, H. (eds) p. 217. Alan R. Liss: New York.